# Personals

**Published:** 1914-08

**Authors:** 


Dr. R. C. Conklin has moved from Batavia to Buffalo.

---

Dr. Lester I. Levyn of Buffalo sailed for Europe early in July.

---

Dr. Douglas P. Arnold of Buffalo returned from Europe, late in June.

---

Jacob Diner of N. Y. has been appointed to the State Board of Pharmacists.

---

Dr. C. A. Potter has been appointed Superintendent of the Gowanda State Hospital.

---

Dr. J. Thornton Barnsdall of Buffalo spent the last half of July fishing in Pennsylvania.

---

Dr. George W. Cottis of Jamestown has been elected a charter fellow of the American College of Surgeons.

---

Dr. Frederick Hayes of Buffalo returned the last of June from a hunting and fishing trip in the Adirondacks.

---

Dr. Solomon C. Martin, Jr., of St. Louis, editor of the "Urologic and Cutaneous Review" called on the Editor in July.

---

Dr. James Wright Putnam of Buffalo has been elected a foreign corresponding member of the Neurologic Society of Paris.

---

Wm. H. Park of N. Y. and Herbert U. Williams of Buffalo have been appointed by the Regents, members of the Medical Council.

---

Mr. J. W. McGee, temporary Food and Drug Inspector for Buffalo, since the resignation of Mr. Dubois, has been recalled to Washington.

---

Dr. Jesse N. Roe announces that he is now located at 392 West Utica Street. Special attention given to dermatology and contagious diseases.

---

Dr. Allen M. Holmes of Buffalo sailed for England, July 11, to attend the International Congress of Surgeons. He will study in Berlin and Vienna before returning.

New appointments to the State Board of Medical Examiners are as follows: Melvin J. Stearns, Ogdensburg; Henry B. Minton, Brooklyn; Hans Zinsser, N. Y.; Wm. G. Bissell, Buffalo; and Ralph H. Williams, D. O., Rochester.

Dr. William Lorenzo Moss, of Baltimore, has been appointed internist to the N. Y. State Institute for the Study of Malignant Disease.

Dr. William Gaertner, of Buffalo, has been appointed a member of the State Medical Advisory Board.

Dr. John A. Ragone, of Buffalo, has opened offices at 18 Irving Place and 290 Swan Street, his practice being limited to diseases of children.

Surgeon-General William C. Gorgas, U. S. A., has received the degree of D. Sc. from Princeton and that of LL. D. from Yale.

Dr. C. J. Smith, of Portland, ex-president of the Oregon State Medical Assn., has been nominated for Governor of his state.

The President of France has conferred upon Dr. Simon Flexner, the cross of Chevalier of the Legion of Honor.

